# The Causes of Acquired Insanity

**Published:** 1913-07

**Authors:** J. Cheston King

**Affiliations:** Atlanta, Ga.


### THE CAUSES OF ACQUIRED INSANITY.

By J. Cheston King, A. B., M. D., Atlanta, Ga.

We see in the history of the medical science to-day the grotesque forms of knowledge, the extinct creation of observant, thoughtful men; in the light we view them to-day, seeing how unfit they are to be associated with the forms and conditions of Knowledge, we at once conclude that they were not only shortcomings but errors, like unto species decaying and dying out; deflections from the true route of progress.

But in that day and time, they served as a complete basis for science, they were as parts of an organic system, complete for that time and potent for a better future. To those who possessed them, they were right, even as to ourselves much of our Knowledge is; though a century from now, truths that seemed complete will be like extinct or monstrous forms and happy should we be if some of the doctrines we enunciate from this notable meeting, called together under the auspices of The Chicago Progressive Medical Society—in whose ranks are to be found men whose influence reaches far beyond the time and space of their conscious activity, will remain in science with the same meaning as they now bear.

Even if our labors in our special line of work have been like waves in advance of the on-coming tide, we are recompensed. A few may watch them and think them grand and beautiful, but they may break on the shore in what seemed like only trouble and confusion, and the tide passes over them and hides the treasures they have borne. But another generation may come and uncover these treasures and worthily display them.

Even to this day and time every writer on acquired insanity has suggested a special classification of the forms, founded upon the ætology or symptomatology of the disease; and the numberless classifications founded on psychological considerations that have been advanced differ more in terminology than in principle and when analyzed are reducible to the principal forms of Hippocrates.

Mania, Melancholy and Dementia.

Dr. J. Batty Tuke adopts the following system based upon the proposition of Morel, which marked a great advance in the study of insanity:

| | |
|---|---|
| (1.) Idiopathic Insanity. | Idiopathic Mania.<br>Melancholia and Dementia.<br>General Paralysis of the Insane. |
| (2..) Traumatic Insanity. | |
| (3.) The Insanities Associated with other Neuroses. | Epileptic Insanity.<br>Hysterical Insanity.<br>Hypochondriacal Insanity. |

(4.) Insanity resulting from the presence of adventitious products.

| | |
|---|---|
| (5.) Insanities resulting from morbid conditions of the general system. | Phthisical Insanity.<br>Rheumatic Insanity.<br>Gouty Insanity.<br>Syphilitic Insanity.<br>Insanity from Sunstroke.<br>Anaemic Insanity.<br>Senile Insanity. |
| (6.) Insanities occurring at evolutional periods. | Climacteric Insanity.<br>Insanity of Pregnancy.<br>Puerperal Insanity. |
| (7.) Toxic Insanity. | Alcoholism, Morphinism, Plumbism, Hydrargyrism, Cocainism, Absynthism, and in fact all those drugs and agents, which induce structural alterations or perversions of the cerebro-spinal system. |

I have after critical study and analysis been able to refer those cases of acquired insanity, which have been under my professional care, to the following causes:

(1.) Hereditary constitution of the cerebro-spinal nervous system, induced by intemperance, syphilis, and other causes acting upon one or both parents. Hereditary insanity, implying hereditary weakness of the nervous system, generally comes on without the interruption of appreciable exciting causes. The nervous system seems to be peculiarly liable to be involved in the effect of hereditary degeneracy, and this is frequently evinced by the occurrence of mental symptoms at those periods of life where either rapid structural development takes place, special functional altering is and was exhibited or is ultimately arrested or upon the advent of senile decay.

We have then an insanity of pubescence, a climateric insanity and a senile insanity. Idiocy and imbecility are frequent results of hereditary weakness, showing itself during foetal life or during the period of detention. The insanity which often affects women at parturition, insanity of pubescence, climateric and senile insanity may often be traced to hereditary weakness of the nervous system.

### MASTURBATION.

The excitement incident to the habitual and frequent indulgence of the unnatural practice of masturbation leads to the most serious constitutional effects and in some cases, to the loss of the higher moral feelings, entire degradation of the moral sense and hopeless insanity. The effects of masturbation are more especially manifested in the nervous system, the functions of which are more or less prevented. The mental faculties become more or less affected, there is great despondency, loss of memory, irritability, irregular action of the heart, derangement of digestion, prostration of strength, neuralgic pains. There is a general loss of health and strength, chronic hypochondriacal invalidism, epileptic seizure, ending in many cases in hopeless insanity.

Guardians, parents and teachers cannot be too careful in guarding those entrusted to their care from this most degrading and pernicious of all habits.

The effects of masturbation in inducing insanity may be witnessed in its victim long after the cessation of the pernicious practice.

### ALCOHOLIC POISONING.

(3.) Alcoholic insanity is met with in three forms:

1. Acute Alcoholic Insanity.
2. Chronic Alcoholic Insanity.
3. Delirium Tremens.

Acute alcoholic insanity seldom occurs except when there is a strong hereditary tendency to mental disturbance, or when the cerebral energies have been notably impaired by excesses or overwork. When all the predisposing causes exist it may not require a large dose of alcohol to bring on an attack. The most frequent form of the affection is violent maniacal delirium, known as mania a potu, with a tendency to homicidal acts.

In some cases the mental disorder takes the melancholic form, and it becomes necessary to guard aainst the strong suicidal tendency.

Chronic alcoholic insanity is one of the results of chronic alcoholism and illustrates in a forceful manner the solidarity of the physical and somatic functions of the nervous system and the interdependance of their morbid manifestations. The mental symptoms are generally all present from the beginning. The sleeplessness so characteristic of commencing disorder is an early symptom; then restlessness and depression with suicidal tendency, sometimes passing rapidly into complete dementia, but generally passing through a course of moral and mental degradation, which progresses step by step, with the symptom of failure of the physical nervous power. Chronic alcoholic insanity presents many points of resemblance to general paralysis of the insane, and is in some cases only to be distinguished from it by the presence of mental depression, which is seldom more than a transitory symptom in the general paralysis.

Delirium Tremens. It is important to note that after the acute symptoms have passed away, in some cases there is left behind a state of sub-acute insanity of characteristic nature. At first suicidal symptoms are apt to appear, suspicions of poisoning, fear of impending evil and hallucinations of hearing. The ordinary vinic or ethyl alcohol in any and every shape, as a sufficient cause of alcoholic insanity is beyond doubt. The more concentrated the alcohol is taken, the more surely and rapidly are its characteristic effects induced; and although some beverages gives a greater liability to certain forms of disease than to others, yet the ultimate tissue changes produced by all are practically similar and of a markedly degenaret character. Chronic alcoholic drinking is undoubtedly hereditary in many cases; not that the ancestors have been necessarily drunkards, but that the family is of an unstable nervous organization, and that the neurotic trait, which shows itself in other members in such affections as epilepsy, hysteria, insanity, is manifested in these cases by an intense craving for alcohol. Sometimes a pernicious education by fostering habits of indulgence in early youth has led to subsequent excess and chronic alcoholism; and the injudicious prescribing of stimulants has occasionally been productive of similar harm. It is well known to pathologists that a large amount of ardent spirits acts on the nerve centers as a narcotic poison and causes rapid death by coma, small quantities produce intoxication accompanied with or followed by an acute congestion and catarrh of the alimentary canal, especially of the stomach and duodenum. Habitual dram drinking by altering the chemical composition of the blood and checking the normal changes of its corpuscles, excites an injurious influence on the nutrition of the tissues. This is increased by the lessened consumption of food and the alteration in the caliber of the blood vessels, set up at first by a special action on the vasomotor nerves, and afterwards maintained by degeneration of their coats, as well as frequently of the heart itself. Alcohol interferes directly with the nutrition of the cell elements of the various organs, including the cerebro-spinal system, as it circulates through them and it retards the elimination of effete materials, carbonic acid, uric acid and urea. In chronic alcoholism the amount of fat in the blood is increased. Chronic congestion and catarrh of the stomach leading to atrophy of the glands and an increase in the sub-mucous connective tissue is very common. The liver is at first enlarged from congestion and may continue so from a subsequent infiltration of fat, but more frequently it shrinks away to cirrhosis. Labor emphysema, chronic bronchitis and hypostatic pneumonia are common. The heart is flabby, dilated and prevents fatty infiltration, and even degeneration of its muscular tissue, but it may be hypertrophied probably as a result of coexistent disease of the kidneys. The arteries and endocardium are studded with other small deposits, the capillaries are congested and the veins varicose. The kidneys exhibit the fatty commonly, the granular form of Brights Disease. The muscles are pale and flabby, the formation of fat takes place at the expense of the bony texture. The nerve centers are atrophied and tough, the convolutions are shrunken, the nerve cells and nerve fibers are wasted and an increased amount of serous fluid exists in the ventricles and sub-arachmoid space. The abnormal adhesion of the dura mater to the cranium, the large Pacchionian bodies, the opaque arachmoid, and the thickened pia-mater, all testify to an aggravated development of fibrous tissue.

For the above reasons our alms-hosues, prisons, hospitals and asylums are filled with diseased, suffering and often incurable human beings.

My last and most important cause for acquired insanity is due to the effect of the action of the poison of syphilis. Insanity often results from the degeneration of the glanglionic cells, nerve tissues and from the formation of gummatous tumors upon the various portions of the cerebro-spinal nervous system. When constitutional syphilis affects the brain and nervous system, the mental symptoms that arise are found, in the majority of cases, to present marked similarity in their character. The mental disturbance is generally preceded by distressing sleeplessness; this is followed by increasing depression of mind. Religious anxiety of a peculiarly hopeless character frequently shows itself. The felling of alarm which accompanies these symptoms sometimes develops into a violent excitement, which may be called maniacal. The development of gummy products within the cranium is frequently evinced by symptoms similar to those of general paralysis. Headache of persistent character, giddiness, vertigo and epiptoid fits occur, accompanied at first with mental depression.

5. Epilepsy—Congenital and Hereditary.

6. Religious Excitement.

The contemplation of certain hypotheses and dogmas held and vehemently urged from the pulpit by some religious sects, have without doubt, produced great excitement and alarm in the minds of persons of excitable and unstable nervous organization. The burning eloquence and moral pictures of the religious enthusiast and fanatic, and the horrible revelation of the melancholy and sinister imagination of Dante, have converted the souls of the unwary and timid into indescribable forebodings and alarm. Certain dogmas, often represented and illustrated by fiery language and by the subtle power of the painter's brush, as the fires and tortures of a burning hell, a veritable lake of fire, whose fiery billows eternally wrapped the bodies and souls of the damned, and whose shores forever resound with the piercing, but hopeless shrieks of those inhabitants of this earth who have failed to enter heaven, on account of the commission of personal sins—veritable living devil, ever on the elert to seduce and damn the souls of men, women and children and drag the unwary down to everlasting confusion and suffering in the bottomless pit.

The violent exercise of certain sects, during the performance of so-called religious exercises, such as shouting, hopping, jumping, dancing, speaking in unknown tongue, often induce epileptic seizure and inaugurate such exhaustion of the nervous structures as induce religious melancholy and end in hopeless insanity.

It is foreign to this paper to dwell upon all the causes of acquired insanity, for I would have to enter into detail upon the effects of race, occupation, climate, age and education. My only incentive is to bring before this honorable body the most prominent causes of acquired insanity, which will result in exciting the public interest in the most distressed and most unfortunate class of fellow citizens, to the end that abuses may be corrected, wrongs righted and the amount of human suffering lessened.

